# Metabolism and Metabolic Reprogramming in Laryngeal Squamous Cell Carcinoma

**DOI:** 10.3390/biomedicines14050959

**Published:** 2026-04-22

**Authors:** Barbara Verro, Roberta Oliveri, Giovanni Pratelli, Marianna Lauricella, Diana Di Liberto, Anna De Blasio, Daniela Carlisi, Carmelo Saraniti

**Affiliations:** 1 Division of Otorhinolaryngology, Department of Biomedicine, Neuroscience and Advanced Diagnostic (BIND), University of Palermo, 90127 Palermo, Italy; carmelo.saraniti@unipa.it; 2 Section of Biochemistry, Department of Biomedicine, Neuroscience and Advanced Diagnostic (BIND), University of Palermo, 90127 Palermo, Italy; roberta.oliveri@unipa.it (R.O.); giovanni.pratelli@unipa.it (G.P.); marianna.lauricella@unipa.it (M.L.); diana.diliberto@unipa.it (D.D.L.); 3 Laboratory of Biochemistry, Department of Biological, Chemical and Pharmaceutical Sciences and Technologies (STEBICEF), University of Palermo, 90127 Palermo, Italy; anna.deblasio@unipa.it

**Keywords:** cancer, larynx, malignant neoplasm, metabolic networks and pathways, Warburg effect, Oncologic

## Abstract

Laryngeal squamous cell carcinoma (LSCC) remains a major clinical challenge within head and neck oncology, with five-year survival rates showing minimal improvement over recent decades despite advances in surgical and multimodal therapeutic strategies. Increasing evidence identifies metabolic reprogramming as a central driver of tumor progression, therapeutic resistance, and immune evasion in LSCC. Beyond the classical Warburg effect, LSCC exhibits profound metabolic reprogramming, involving coordinated alterations in carbohydrate, amino acid, lipid, and iron metabolism that support adaptation to hypoxic and nutrient-deprived microenvironments. Hypoxia-inducible factors, particularly HIF-1α, coordinate these key biochemical pathways and enzymatic steps by integrating glycolysis, glutaminolysis, folate-dependent one-carbon pathways, lipid synthesis, and mitochondrial remodeling, while also influencing stromal and immune components of the tumor microenvironment. Metabolic crosstalk between tumor cells, cancer-associated fibroblasts, and immune populations promotes immunosuppression through nutrient competition and accumulation of metabolites such as lactate and lipid-derived mediators. In parallel, dysregulated iron handling and altered ferroptosis susceptibility emerge as key determinants of tumor aggressiveness and treatment response. This review synthesizes current evidence on metabolic rewiring in laryngeal squamous cell carcinoma, highlighting how alterations in metabolic pathways create targetable vulnerabilities that drive tumor biology, immune modulation, and resistance to conventional and emerging therapies. Elucidating these metabolic dependencies may support the development of metabolism-based biomarkers and therapeutic strategies in laryngeal squamous cell carcinoma, providing an integrated and translational perspective that links tumor metabolism with microenvironmental interactions and immune modulation, while highlights emerging therapeutic vulnerabilities.

## 1. Introduction

Laryngeal carcinoma represents one of the most common neoplasms of the head and neck region, with substantial morbidity and a profound impact on patients’ quality of life. Despite refinements in organ-preserving surgical techniques and significant advances in radiotherapy and chemoradiotherapy protocols, overall five-year survival rates for laryngeal squamous cell carcinoma (LSCC) have remained largely unchanged over the past three decades, stabilizing at approximately 60% [1]. This persistent survival plateau underscores an urgent need for improved prognostic biomarkers and the identification of novel therapeutic targets capable of addressing disease progression and treatment resistance. The biological complexity of LSCC reflects the convergence of unique anatomical features of the larynx with well-established environmental risk factors, most notably tobacco smoking and alcohol consumption, which together drive carcinogenesis [2]. From a molecular perspective, LSCC does not represent a single homogeneous entity but rather arises from the progressive accumulation of genetic and epigenetic alterations that disrupt key regulatory pathways controlling cell proliferation, differentiation, and survival. Early molecular profiling studies have demonstrated that this heterogeneity significantly influences clinical behaviour, prognosis, and responsiveness to conventional therapies [3]. However, while genomic and proteomic analyses have provided valuable catalogues of mutations and altered signalling pathways, they offer only a partial view of tumor biology. Less attention has been paid to how these molecular alterations are functionally translated into adaptive cellular programs that allow tumor cells to survive and thrive under adverse conditions.

In this context, metabolic reprogramming has emerged as a fundamental hallmark of LSCC. Tumor growth within the larynx often occurs in a microenvironment characterized by irregular vascularization, fluctuating oxygen availability, and limited nutrient supply. To sustain proliferation and survival under these constraints, LSCC cells undergo extensive metabolic remodeling that extends well beyond enhanced glycolysis [2]. This metabolic rewiring encompasses coordinated alterations in energy production, biosynthetic pathways, redox homeostasis, and metabolite signaling, with far-reaching consequences for epigenetic regulation, immune evasion, and resistance to therapy [3].

While current clinical management of LSCC emphasizes organ preservation and multidisciplinary treatment strategies, a deeper understanding of tumor metabolism offers an additional and potentially transformative therapeutic perspective. This review aims to examine the biochemical and molecular mechanisms through which LSCC cells reprogram their metabolism to promote survival and chemoresistance. By integrating anatomical, molecular, and clinical perspectives, we explore how metabolic reprogramming may represent an “Achilles’ heel” of LSCC, as originally conceptualized in cancer biology, offering new opportunities for the development of targeted therapies and next-generation biomarkers [4]. Unlike previous studies that have largely addressed Head and Neck SCC (HNSCC) as a homogeneous group, this review provides a focused and translational perspective linking tumor metabolism with tumor microenvironment (TME) interactions and immune modulation, while emphasizing clinically relevant biomarkers and emerging therapeutic strategies specific to LSCC.

## 2. Metabolic Reprogramming in Cancer and LSCC

Metabolic reprogramming represents a dynamic and tightly regulated adaptive process that enables cancer cells to cope with environmental stress while sustaining proliferation and survival. Rather than reflecting a simple shift in energy production, metabolic rewiring integrates nutrient sensing, oxygen availability, and intracellular signaling to modulate bioenergetic, biosynthetic, and redox pathways. Importantly, this process extends beyond malignant cells and involves complex interactions with stromal and immune components of the TME, collectively shaping tumor behaviour and therapeutic response [5].

Beyond classical paradigms such as the Warburg effect and mitochondrial oxidative phosphorylation, contemporary studies demonstrate that cancer cells retain functional mitochondria and can flexibly transit between metabolic states in response to local conditions. This plasticity allows tumors to adapt to fluctuations in oxygen tension, nutrient availability, and pH, conferring a selective advantage in poorly perfused and hypoxic regions. Cancer-associated fibroblasts (CAFs) actively participate in this metabolic remodeling by adjusting glycolysis, lipid metabolism, and anabolic processes to support tumor growth, facilitate immune evasion, and promote resistance to therapy. Through metabolite exchange and paracrine signaling, CAFs help establish a metabolically supportive niche that reinforces tumor cell survival [6]. Metabolic reprogramming also exerts profound effects on antitumor immunity. Altered nutrient fluxes and metabolite accumulation within the TME impose metabolic constraints on immune effector cells, including cytotoxic T lymphocytes and natural killer (NK) cells, limiting their proliferation and function. At the same time, metabolites such as lactate, adenosine, and products of amino acid metabolism act as immunosuppressive signals, promoting the expansion of regulatory T cells and other suppressive immune populations. These combined effects generate an immunologically permissive microenvironment that favors tumor persistence and progression [7].

Taneja et al. propose hypoxia-inducible factor 1 (HIF-1α) as a key regulator of tumor metabolic plasticity, capable of coordinating energy metabolism, biosynthesis, adaptation to hypoxia, and modulation of the microenvironment [8]. Indeed, under hypoxic conditions, HIF-1α orchestrates a broad transcriptional program that regulates glycolytic enzymes, nutrient transporters, and mitochondrial function, HIF-1α enables cancer cells to maintain adenosine triphosphate (ATP) production while limiting oxidative stress. In parallel, HIF-1α influences non-metabolic processes, including invasion, metastatic dissemination, acquisition of stem-like properties, and resistance to chemotherapy, underscoring its role as an integrative driver of malignant progression.

From a therapeutic perspective, the dependencies created by metabolic rewiring offer attractive opportunities for targeted intervention. Inhibiting key metabolic enzymes, disrupting nutrient sensing pathways, or interfering with stromal tumor and immunometabolic crosstalk may selectively compromise tumor viability while enhancing antitumor immune responses. In particular, targeting metabolic interactions between cancer cells and CAFs, as well as metabolites that promote immunosuppression, represents a promising strategy to overcome therapeutic resistance.

LSCC exhibits pronounced metabolic reprogramming that mirrors and, in some aspects, accentuates these general cancer-associated adaptations. Beyond classical alterations such as the Warburg effect and mitochondrial oxidative phosphorylation, LSCC cells exploit a wide array of metabolic pathways—including glycolysis, glutaminolysis, lipid synthesis, and one-carbon metabolism—to adapt to hypoxic and nutrient-limited microenvironments. Hypoxia-inducible factors (HIFs), particularly HIF-1α, orchestrate many of these adaptations by promoting glycolytic flux, redirecting α-ketoglutarate toward reductive carboxylation for lipid synthesis, and preserving intracellular glutamine for nucleotide, amino acid, and lipid biosynthesis. Similarly, upregulation of serine metabolism fuels the mitochondrial folate-dependent one-carbon cycle, enhancing Nicotinamide Adenine Dinucleotide Phosphate (NADPH) production for redox homeostasis under oxygen deprivation and supporting nucleotide biosynthesis [9]. These metabolic adaptations are tightly linked to modulation of the immune microenvironment. Tumor cells compete with infiltrating immune cells for nutrients, while simultaneously releasing metabolites that shape immunosuppressive niches. CAFs and other stromal components actively participate in this crosstalk, providing metabolic support and promoting immune evasion. Ma et al. emphasize that these interactions contribute to resistance against conventional therapies and immunotherapy, underlining the need to integrate metabolic targeting into clinical strategies [9]. Importantly, LSCC displays distinct metabolic subtypes with prognostic significance. Zheng et al. identified two molecular subgroups based on metabolic gene expression: one associated with favorable outcomes and enriched in amino acid, lipid, and vitamin metabolism, and another linked to poor prognosis, characterized by increased immune checkpoint activity, DNA methylation, and aggressive metabolic phenotypes. This stratification suggests that metabolic profiling could inform patient-specific therapeutic approaches [10]. Finally, mitochondrial metabolism emerges as a critical determinant of LSCC progression. Hou et al. report that mitochondria-driven pathways, including glutamine utilization, fatty acid oxidation (FAO), and tricarboxylic acid (TCA) cycle remodeling through reduced pyruvate oxidation and enhanced glutamine-dependent anaplerosis, sustain tumor growth while promoting chemoresistance and adaptation to hypoxia [11]. Targeting these mitochondrial dependencies may provide novel therapeutic opportunities, either alone or in combination with metabolic and immune-based interventions [11]. Together, these findings highlight the multifaceted nature of metabolic reprogramming in LSCC, encompassing bioenergetic, biosynthetic, and immunoregulatory processes. The following sections examine how specific metabolic pathways are altered in LSCC and how these alterations contribute to tumor progression and therapeutic resistance.

## 3. Carbohydrate Metabolism

Carbohydrate metabolism, encompassing glycolysis, the TCA cycle, and the pentose phosphate pathway (PPP), forms the metabolic backbone of cancer cells, providing both energy and essential biosynthetic precursors. Glycolysis, in particular, serves as a rapid energy-generating pathway by converting glucose into pyruvate while producing ATP and Nicotinamide Adenine Dinucleotide (NADH). Thus, carbohydrate metabolism in cancer cells, and in particular the phenomenon known as the Warburg effect, represents a cornerstone of metabolic reprogramming in cancer. Historically interpreted as an irreversible defect in mitochondrial oxidative phosphorylation, the Warburg effect is now redefined as a selected adaptive strategy to support the growth and survival of neoplastic cells in hostile microenvironments. As discussed by Vaupel and colleagues, aerobic glycolysis does not reflect mitochondrial dysfunction, but rather an actively selected metabolic strategy during tumor progression [12]. Neoplastic cells maintain a functional oxidative capacity, integrating mitochondrial energy production with increased glycolytic flux, in response to the unfavourable environmental conditions typical of the tumor microenvironment. A central element of this reconsideration is the role of chronic and intermittent hypoxia. Disorganized tumor growth and irregular vascularization generate oxygen gradients that impose high metabolic flexibility on cells. The authors emphasize that tumor metabolism must be considered as a dynamic and highly plastic system, capable of rapidly adapting to the fluctuations in oxygen, nutrients, and pH that characterize the neoplastic microenvironment. In this context, the coexistence of aerobic glycolysis and oxidative phosphorylation represents an evolutionarily advantageous survival strategy, which allows tumor cells to modulate their energy structure as a function of local conditions rather than adhering to a single rigid metabolic program. Thus, increased glycolysis allows tumor cells to ensure rapid and relatively oxygen-independent energy production, promoting survival in poorly perfused regions. Activation of hypoxia-sensitive transcription factors, particularly HIF-1α, promotes the expression of glycolytic enzymes and glucose transporters (e.g., GLUT1 and GLUT3), thereby supporting oxygen-independent ATP production. In parallel, HIF-1α suppresses mitochondrial function by promoting inhibition of pyruvate entry into the TCA and reducing Oxidative Phosphorylation (OXPHOS) activity, thereby limiting the production of potentially toxic mitochondrial Reactive Oxygen Species (ROS) under hypoxic conditions [8]. High glycolytic flux fuels collateral anabolic pathways, such as the PPP, contributing to nucleotide synthesis and maintaining redox balance through NADPH production. In this way, aerobic glycolysis supports not only cell proliferation but also protection from oxidative stress [13], an aspect particularly relevant in a microenvironment characterized by metabolic instability. Cancer cells often exhibit upregulated expression of key PPP enzymes, particularly glucose-6-phosphate dehydrogenase (G6PD), resulting in an increased flux through this pathway. The activity of G6PD is tightly regulated by multiple oncogenic signals: transcription factors such as c-Myc and TAp73, as well as the PI3K-AKT signaling pathway, can enhance PPP flux either by promoting G6PD transcription or by stabilizing the enzyme [14,15]. This upregulation not only ensures a continuous supply of NADPH for redox balance and lipid synthesis but also provides ribose sugars necessary for nucleotide and macromolecule synthesis, supporting tumor growth, survival, and adaptation to metabolic stress [5].

The study by Dessì et al., published in 1990, provides the only detailed investigation of G6PD activity in patients with LSCC [16]. The researchers examined enzyme activity in both normal and tumor laryngeal tissues from individuals with and without G6PD deficiency, a genetic condition characterized by reduced enzymatic function and increased susceptibility to oxidative stress. Using spectrophotometric assays to measure the conversion of NADP^+^ to NADPH, they compared enzymatic activity between tumor and adjacent normal tissues. Remarkably, their results showed that G6PD activity was significantly higher in tumor tissues than in normal tissues across both G6PD-normal and G6PD-deficient patients (*p*-value < 0.05). Even in subjects with systemic G6PD deficiency, whose normal tissues typically display minimal or undetectable activity, tumor tissues exhibited a clear upregulation of G6PD. This suggests that LSCC cells can activate compensatory metabolic pathways to overcome inherited enzymatic limitations, ensuring sufficient NADPH supply for anabolic processes and defence against oxidative stress. The authors interpreted these data as an indication that laryngeal carcinoma can increase G6PD activity even in the presence of a genetic deficiency, probably to meet the NADPH requirement necessary for biosynthetic processes and defence against oxidative stress. This contribution, although dated, paved the way for the hypothesis that G6PD may play a relevant role in the biology of LSCC and deserves further investigation as a possible therapeutic target.

A central aspect discussed by Vaupel and Multhoff concerns the role of lactate, which is reevaluated from a simple metabolic byproduct to a true signal metabolite. Lactate contributes to the creation of an acidic microenvironment that promotes invasion, angiogenesis, and immunosuppression, but can also be reabsorbed and used as an energy source by more oxygenated tumor or stromal cells. Beyond its intracellular roles, lactate exerts significant effects on surrounding cells, particularly immune populations. Accumulation of lactate in the TME modulates immune cell function, driving macrophage polarization toward pro-tumor phenotypes, a process supported by ATP-citrate lyase (ACLY) and mitochondrial pyruvate metabolism [17]. Additionally, lactate influences T cell activity through both pH-dependent and pH-independent mechanisms, including metabolic rewiring, which can impair anti-tumor immune responses and facilitate immune evasion [18]. Collectively, these findings highlight lactate as a central mediator of tumor progression, linking glycolytic metabolism to immune modulation, angiogenesis, and stromal remodelling.

This concept of “metabolic symbiosis” challenges the idea of uniformly glycolytic tumor metabolism, highlighting instead a metabolic cooperation between different cell populations within the tumor [19].

In LSCC, one of the most prominent consequences of enhanced glycolytic metabolism is the accumulation of lactate within the TME. This metabolic byproduct is not merely a waste product of glycolysis but functions as a potent modulator of the immune landscape in the TME. The review by Ma et al. [9] highlights that metabolomic analyses of LSCC tissues have repeatedly identified elevated lactate as a key altered metabolite, reflecting both increased glycolytic flux and downstream metabolic rewiring in tumor cells. This abnormal lactate accumulation has profound effects on immune cell function in LSCC; it can impair the cytotoxic activity of CD8^+^ T cells and NK cells, two critical effectors of antitumor immunity, while simultaneously promoting the differentiation and function of Tregs cells, which suppress effector T cells’ immune responses. Taken together, these lactate-driven alterations illustrate how metabolic reprogramming in LSCC extends beyond intrinsic tumor cell energetics to actively reshape the immune contexture of the TME, reinforcing immune evasion and reducing the efficacy of immunotherapeutic strategies. Targeting lactate production or its downstream effects on immune cells thus represents a compelling avenue for enhancing antitumor immunity in LSCC, particularly when combined with immune checkpoint blockade or other immunomodulatory treatments.

**Figure 1:**
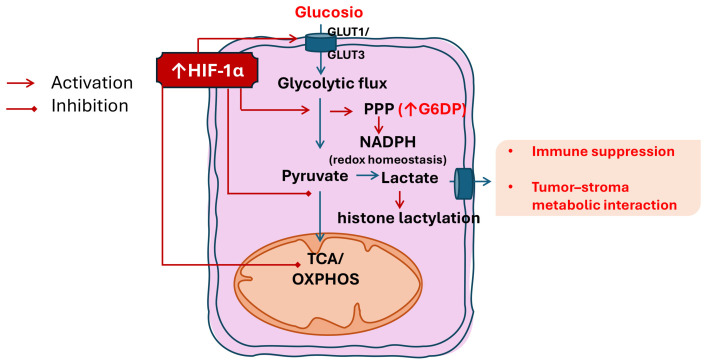
Carbohydrate metabolic reprogramming in laryngeal squamous cell carcinoma (LSCC). Under hypoxic conditions, LSCC cells increase glucose uptake and glycolytic flux through HIF-1α–dependent mechanisms. HIF-1α limits pyruvate entry into the tricarboxylic acid (TCA) cycle, thereby reducing mitochondrial oxidative phosphorylation (OXPHOS) without complete loss of mitochondrial function and favoring the conversion of pyruvate into lactate. Enhanced diversion of glycolytic intermediates into the pentose phosphate pathway (PPP) supports NADPH production and redox homeostasis, with increase. Lactate accumulation within the tumor microenvironment acts as a signaling metabolite, promoting immune suppression and metabolic symbiosis between tumor and stromal cells. Lactate also contributes to epigenetic regulation through histone lactylation, supporting sustained transcriptional programs associated with tumor adaptation and immune modulation. These adaptations support tumor survival but also create therapeutic opportunities by sensitizing LSCC cells to oxidative stress–based therapies and immunomodulatory strategies.

In addition to its extracellular signalling functions, lactate serves as a substrate for post-translational modifications, including protein lactylation, thereby linking metabolic reprogramming to epigenetic and transcriptional regulation. Lactylation occurs through the formation of a peptide bond between a lactyl-CoA group and the ε-amino group of lysine residues on both histone and non-histone proteins. Notably, histone lactylation, such as histone H3K18 lactylation (H3K18la), has been implicated in the aberrant activation of oncogenic transcriptional programs [20]. In a 2025 study, Fu et al. demonstrated that H3K18la epigenetically regulates the expression of Runt-related transcription factor 2 (RUNX2)—a master regulator of osteogenesis that functions as an oncogenic driver in multiple malignancies—promoting LSCC progression through activation of the PI3K/AKT signalling pathway [21]. These findings deepen our molecular understanding of LSCC pathogenesis and identify the H3K18la-RUNX2 axis as a critical regulatory mechanism in cancer biology. Furthermore, exploring potential crosstalk between the H3K18la-RUNX2 axis and other signalling pathways involved in LSCC progression may offer new insights into the disease’s pathogenesis and therapeutic strategies (Table 1) (Figure 1).

## 4. Amino Acid Metabolism

Amino acid metabolic reprogramming is a central feature of cancer cell adaptation, enabling tumor cells to meet the heightened demands for energy, nucleotides, redox balance, signalling, and epigenetic regulation. Tumor cells alter the metabolism of key amino acids not only to fuel proliferation and biosynthesis but also to modulate the TME. As highlighted by Liu et al. [22], amino acids can act as signalling molecules, influencing immune cell activity, promoting immune evasion, and reshaping anti-tumor responses. Moreover, the reprogramming of amino acid metabolism affects stromal components, including CAFs, drives extracellular matrix remodelling, and supports angiogenesis, collectively facilitating tumor growth and metastasis. These insights underscore the dual role of amino acid metabolism: sustaining intrinsic tumor cell survival while simultaneously orchestrating TME dynamics that enhance immune suppression and therapeutic resistance. Understanding these interactions offers potential avenues for targeted interventions that disrupt metabolic dependencies and restore anti-tumor immunity.

Among these, glutamine plays a central role as both an energy source and a biosynthetic precursor. Glutamine is converted into glutamate by glutaminase (GLS), and subsequently into α-ketoglutarate (α-KG), which feeds into the TCA cycle, integrating glutamine as a critical nitrogen and carbon donor for rapid cellular proliferation [23]. In hypoxic conditions, HIF-1α orchestrates a metabolic shift from oxidative metabolism to reductive carboxylation, redirecting α-ketoglutarate toward isocitrate and citrate through a “reverse” TCA cycle. This metabolic rewiring supports the synthesis of acetyl-CoA and lipids essential for cellular maintenance and proliferation under low-oxygen conditions. Mechanistically, HIF-1α promotes the ubiquitination and proteasomal degradation of the E1 subunit of the α-ketoglutarate dehydrogenase (αKGDH) complex, primarily via the E3 ligase Seven In Absentia Homolog 2 (SIAH2). As αKGDH is required for the oxidative decarboxylation of α-ketoglutarate, its inhibition limits glutamine-derived α-ketoglutarate oxidation. Rather than increasing glutamine synthesis, this process limits glutamine catabolism, thereby preserving intracellular glutamine and its carbon skeletons for alternative biosynthetic pathways. Consequently, glutamine becomes available to sustain reductive TCA flux, nucleotide biosynthesis, and the synthesis of amino acids and fatty acids. In addition, glutamine contributes to the production of uridine diphosphate N-uridine diphosphate N-acetylglucosamine (UDP-GlcNAc), supporting protein folding, glycosylation, and intracellular trafficking, thus ensuring cellular homeostasis and biosynthetic capacity in hypoxic TME [24]. Beyond its role in energy production, glutamine provides nitrogen atoms via transaminase reactions, supporting nucleotide and amino acid synthesis [24]. Additionally, in tumor cells with activated KRAS signalling, glutamine metabolism contributes to the maintenance of intracellular redox homeostasis, ensuring a balance between ROS and antioxidant capacity, which is essential for cell survival under metabolic stress.

Asparagine (Asn) plays a dual role in cancer. It supports tumor cell survival and proliferation, being synthesized from aspartate (Asp) via asparagine synthetase (ASNS), with aspartate representing a key metabolic limiting factor [25]. While direct evidence linking asparagine metabolism to LSCC is currently lacking, studies in other tumor models indicate that asparagine availability critically regulates both tumor cell proliferation and CD8^+^ T cell–mediated antitumor immunity. Beyond tumor cells, Asn influences immune responses: in CD8^+^ T cells, it can enhance T cell receptor signalling via the lymphocyte-specific protein tyrosine kinase (LCK), promoting anti-tumor activity [26]. However, the effects of Asn are context- and time-dependent: restriction of Asn can suppress early T cell activation but later promote differentiation and Nuclear Factor Erythroid 2–Related Factor 2 (NRF2)-mediated anti-tumor responses [27]. Overall, Asn availability exhibits a complex role, supporting tumor growth while simultaneously modulating T cell-mediated immunity.

Serine plays a central role in supporting cancer cell growth by contributing to multiple biosynthetic and regulatory processes. Beyond its function as a building block for phospholipids such as phosphatidylserine, serine serves as a precursor for glycine and cysteine synthesis and represents a major source of one-carbon units for the folate cycle. Under hypoxic conditions, HIFs transcriptionally regulate key components of serine metabolism and the mitochondrial one-carbon pathway. In particular, HIF-1α activation induces the overexpression of phosphoglycerate dehydrogenase (PHGDH), the rate-limiting enzyme of the serine synthesis pathway, thereby enhancing intracellular serine production in cancer cells [28,29]. One-carbon metabolism, also referred to as the folate cycle, comprises a network of reactions that transfer one-carbon groups in various oxidation states to sustain nucleotide synthesis, redox balance, and epigenetic regulation. In mitochondria, serine hydroxymethyltransferase 2 (SHMT2) converts serine into glycine while generating 5,10-methylene-tetrahydrofolate. This intermediate is further processed by methylenetetrahydrofolate dehydrogenase 2, producing NADPH. The increase in NADPH strengthens antioxidant defences and helps cancer cells cope with oxidative stress [30]. Overall, HIF-1α-driven activation of serine and one-carbon metabolism supports cancer cell survival and proliferation, especially in hypoxic environments.

**Figure 2:**
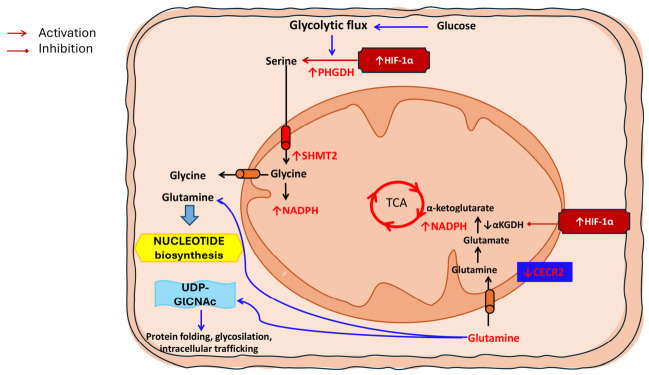
Amino acid metabolic reprogramming in laryngeal squamous cell carcinoma (LSCC). Amino acid metabolism is extensively rewired in LSCC to support biosynthesis, redox homeostasis, and adaptation to hypoxic stress. Enhanced glycolytic flux fuels the serine synthesis pathway through hypoxia-inducible factor-1α (HIF-1α)–mediated upregulation of phosphoglycerate dehydrogenase (PHGDH), while increased mitochondrial serine hydroxymethyltransferase 2 (SHMT2) activity promotes glycine production and nicotinamide adenine dinucleotide phosphate (NADPH) generation. Glutamine acts as a central metabolic hub, feeding the tricarboxylic acid (TCA) cycle via conversion to glutamate and α-ketoglutarate. Under hypoxic conditions, HIF-1α suppresses α-ketoglutarate dehydrogenase (αKGDH), promoting TCA rewiring and preserving glutamine-derived carbon skeletons for anabolic pathways, including nucleotide biosynthesis and UDP-N-acetylglucosamine (UDP-GlcNAc) production. Loss of the tumor suppressor CECR2 further enhances glutamine utilization, reinforcing mitochondrial adaptation and anabolic growth in LSCC.

Amino acid metabolism is a fundamental component of metabolic reprogramming in LSCC, directly contributing to biosynthesis, redox homeostasis, and signalling processes that support tumor growth and survival. Recent comprehensive reviews on mitochondrial metabolism in LSCC underscore that alterations in amino acid utilization are not merely collateral to energy production but represent active, regulated adaptations that interface with mitochondrial function, anabolic pathways, and stress responses. Hou et al. describe how changes in the metabolism of key amino acids—especially glutamine, but also serine and others—feed into mitochondrial pathways that sustain the bioenergetic and biosynthetic needs of LSCC. Specifically, glutamine contributes carbon and nitrogen for the TCA cycle, supports nucleotide synthesis, and helps maintain redox balance through production of NADPH, with these functions becoming particularly important under hypoxic and nutrient-limited conditions common in solid tumors [11]. In LSCC, the reliance on glutamine and other amino acid substrates integrates with mitochondria-mediated adaptations, positioning amino acid metabolism as both a driver of disease progression and a determinant of therapeutic response. The clinical relevance of amino acid reprogramming in LSCC is further highlighted by molecular studies demonstrating direct mechanistic links between specific regulatory proteins and glutamine metabolism. Wang et al. identified CECR2 (Cat Eye Syndrome Chromosome Region, Candidate 2), a novel tumor suppressor, as a key regulator of glutamine utilization in LSCC. Loss of CECR2 expression was shown to enhance glutamine metabolism, promoting glutaminolysis and supporting rapid tumor cell proliferation. Mechanistically, CECR2 downregulation led to increased expression and activity of enzymes involved in glutamine catabolism, thereby elevating glutamine flux into the TCA cycle and downstream biosynthetic routes. This shift not only supplied critical intermediates for anabolic growth but also augmented antioxidant defences by increasing glutamine-dependent production of reducing equivalents. Importantly, restoration of CECR2 expression or pharmacological modulation of glutamine metabolism impaired tumor growth in preclinical models, suggesting that targeting aberrant glutamine utilization may offer therapeutic benefit in LSCC [31] (Table 2) (Figure 2).

## 5. Lipid Metabolism

Lipids are not only crucial for energy production but also serve as fundamental components of cell membranes. Elevated lipid levels therefore support tumor cell proliferation, while simultaneously providing substrates for signaling molecules and membrane remodeling [5]. Consequently, targeting lipid metabolism or lipid-sensing pathways has emerged as a promising therapeutic strategy in various cancers, offering the potential to disrupt tumor growth and survival. Alterations in lipid metabolism represent a crucial aspect of tumor cell reprogramming, supporting both proliferation and metastasis. Lipid metabolic pathways encompass the synthesis and degradation of fatty acids, cholesterol, and lipid droplet (LDs), which serve not only as energy reservoirs but also as building blocks for membrane biogenesis and signaling molecules. In many cancers, lipid metabolism is reprogrammed in favor of fatty acid synthesis, a process driven by the upregulation of key enzymes such as acetyl-CoA carboxylase (ACC) and fatty acid synthase (FASN). The expression of these enzymes is often enhanced through activation of sterol regulatory element-binding proteins (SREBPs), master transcription factors controlling lipid homeostasis [32]. Cancer cells display a profound rewiring of lipid metabolism under hypoxic conditions, largely orchestrated by HIF-1α whose activation promotes LDs accumulation and enhances de novo lipid synthesis, while concomitantly suppressing mitochondrial fatty acid β-oxidation [33]. This shift toward lipid storage rather than lipid catabolism is critical for tumor cell survival, as HIF-1α-driven triacylglycerol synthesis provides readily accessible energy reserves, protects cells from lipotoxic stress, and supports the biogenesis of cellular and organelle membranes required for sustained proliferation [34,35]. Hypoxia also alters the balance between saturated and unsaturated fatty acids, leading to an accumulation of saturated species that can be detrimental to membrane integrity and cell viability. To counteract this potentially toxic effect, HIF-1α induces the expression of key lipogenic enzymes, including FASN and stearoyl-CoA desaturase-1 (SCD1). Notably, SCD1 plays a pivotal role by converting saturated stearate into monounsaturated oleate, thereby restoring an appropriate saturated-to-unsaturated fatty acid ratio. This desaturation process is essential for maintaining membrane fluidity, preserving cellular integrity, and preventing apoptosis [8,36]. In parallel, HIF-1α further reinforces this lipogenic phenotype by actively repressing mitochondrial FAO, consolidating a metabolic state that favours lipid accumulation and tumor cell adaptation to hypoxic stress [37].

Dysregulation of cholesterol metabolism also contributes to tumor progression. In particular, the accumulation of 27-hydroxycholesterol, a circulating cholesterol metabolite, has been shown to promote metastasis by increasing resistance to ferroptosis, a form of programmed cell death dependent on lipid peroxidation [38]. Overall, these alterations in lipid metabolism not only supply energy and structural components necessary for rapid tumor growth but also provide mechanisms for therapy resistance and metastatic potential, highlighting lipid pathways as potential therapeutic targets in cancer treatment.

A foundational perspective on lipid alterations in the head and neck cancer spectrum, which includes LSCC, highlights the upregulation of key lipogenic enzymes and transporters. Enzymes such as FASN and ACC, which catalyze de novo fatty acid synthesis, are frequently overexpressed, facilitating the production of palmitate and other long-chain fatty acids used for membrane biogenesis and energy storage. In addition, fatty acid binding proteins (FABPs) and transporters like CD36 are upregulated, increasing the uptake and intracellular trafficking of fatty acids, while alterations in cholesterol metabolism pathways further contribute to membrane integrity and signaling lipid pools [39]. In LSCC specifically, early work by Louw and Claassen proposed the rationale for modulating fatty acid metabolism as an adjuvant strategy to improve radiotherapy outcomes, arguing that aberrant lipid utilization may underlie radiotherapy failure and tumor recurrence by providing metabolic flexibility and resistance to oxidative stress. Although clinical validation remains limited, this concept underscores the importance of lipid availability and utilization in determining therapeutic responses in early LSCC [40]. More recent reviews of head and neck carcinoma demonstrate that lipid metabolic reprogramming also intersects with inflammatory signaling and immune modulation. Lipid metabolites and their dysregulated synthesis influence the production of pro- and anti-inflammatory mediators within the TME, promoting an environment that can dampen effective antitumor immunity while supporting cancer cell survival. Inflammatory factors linked to aberrant lipid metabolism include various eicosanoids and lipid-derived cytokines that modulate immune cell recruitment and function, potentially contributing to the immunosuppressive milieu observed in LSCC [41].

**Figure 3:**
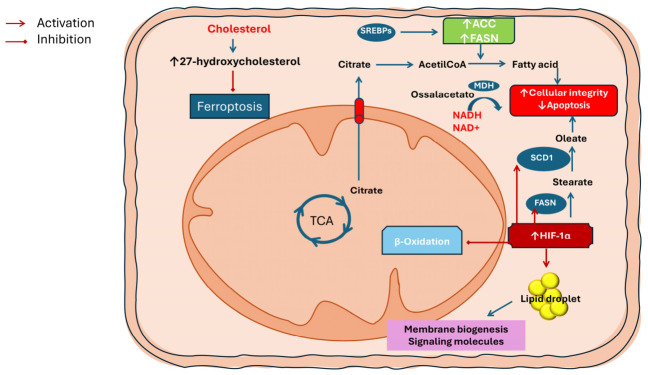
Lipid metabolic reprogramming in laryngeal squamous cell carcinoma (LSCC). Under hypoxic conditions, HIF-1α promotes de novo lipogenesis by enhancing citrate export and activating SREBP-dependent expression of acetyl-CoA carboxylase (ACC) and fatty acid synthase (FASN), while concomitantly suppressing mitochondrial fatty acid β-oxidation. Increased SCD1 activity restores the balance between saturated and monounsaturated fatty acids, preserving membrane integrity and preventing apoptosis. Hypoxia also favors lipid droplet accumulation as energy reserves and promotes resistance to ferroptosis through cholesterol-derived metabolites such as 27-hydroxycholesterol.

These cumulative changes in lipid metabolism not only provide building blocks for membranes and energy reserves but also generate lipid signaling molecules that influence cell proliferation, stress responses, and interactions with stromal and immune cells. The broad dysregulation of both lipid biosynthetic and catabolic pathways observed in LSCC underscores the potential of targeting lipid metabolic nodes—for example, inhibiting FASN or modulating fatty acid uptake—as strategies to weaken tumor growth and enhance sensitivity to existing therapies [39] (Table 3) (Figure 3).

## 6. Iron Metabolism

Iron metabolism is fundamental for both normal and cancer cell physiology, participating in critical processes such as DNA synthesis, DNA repair, and oxygen transport [42]. Cellular iron homeostasis encompasses uptake, utilization, storage, and export, and its dysregulation is a common feature in many cancers, contributing to tumor initiation, growth, and metastasis [43]. Paradoxically, while iron supports tumor proliferation, it also serves as a key mediator of ferroptosis, a form of regulated cell death driven by iron-dependent lipid peroxidation. Elevated intracellular iron levels can sensitize cells to ferroptosis, which may act as a natural barrier to uncontrolled tumor growth [44]. This dual role highlights the complex interplay between iron metabolism and cancer progression, suggesting that modulation of iron homeostasis and ferroptosis could provide novel therapeutic opportunities.

**Figure 4:**
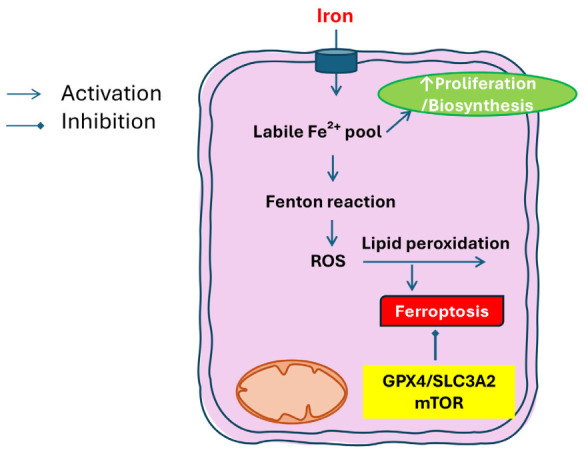
Iron metabolism and ferroptosis in laryngeal squamous cell carcinoma (LSCC). Iron uptake sustains tumor cell proliferation and biosynthetic processes, supporting DNA synthesis, mitochondrial function, and metabolic activity. At the same time, expansion of the intracellular labile Fe^2+^ pool promotes Fenton reactions, reactive oxygen species (ROS) generation, and lipid peroxidation, ultimately triggering ferroptotic cell death. LSCC cells counteract ferroptosis through the activation of antioxidant and nutrient-sensing pathways, including glutathione peroxidase 4 (GPX4), the cystine/glutamate antiporter subunit SLC3A2, and mTOR signaling, thereby preserving redox balance and promoting tumor survival.

Altered iron metabolism and ferroptosis have emerged as central features of metabolic reprogramming in LSCC, with important implications for tumor progression, prognosis, and therapeutic targeting. In this context, ferroptosis represents a nexus between iron handling, oxidative stress, and membrane lipid metabolism, distinct from apoptosis or necrosis. LSCC cells often exhibit dysregulated iron homeostasis, characterized by increased iron uptake, altered storage, and changes in iron efflux pathways. These alterations create a labile iron pool that can catalyze the formation of ROS and sensitize cells to oxidative damage. Because iron is essential for many cellular processes—including DNA synthesis, oxygen transport, and mitochondrial function—tumors adapt their iron metabolism both to support rapid proliferation and to mitigate iron-mediated toxicity. A growing body of evidence highlights ferroptosis as both a vulnerability and a therapeutic opportunity in LSCC. Luo et al. comprehensively review ferroptosis pathways in laryngeal cancer, emphasizing that many LSCC cells upregulate antioxidant systems—such as glutathione peroxidase 4 (GPX4) and components of the cystine/glutamate antiporter—to counteract iron-induced lipid peroxidation and prevent ferroptotic cell death. These adaptations help tumor cells survive under metabolic stress and resist conventional therapies. Conversely, pharmacological induction of ferroptosis, by perturbing iron metabolism or lipid peroxidation defences, has been proposed as a potential non-surgical therapeutic strategy [45]. Several molecular studies support the clinical relevance of ferroptosis regulation in LSCC. Ji et al. identified a panel of ferroptosis-related genes whose expression distinguishes LSCC tissues from normal counterparts and correlates with patient prognosis, suggesting that ferroptosis-associated gene signatures could serve as diagnostic or prognostic biomarkers [46]. Similarly, Han et al. reported that expression levels of specific ferroptosis regulators are linked to survival outcomes in LSCC, reinforcing the idea that ferroptotic propensity influences tumor aggressiveness and therapy responsiveness [47]. Mechanistic insights into ferroptosis resistance have also been provided by functional studies. Wu et al. demonstrated that Solute Carrier Family 3 Member 2 (SLC3A2), a component of the cystine/glutamate antiporter system, inhibits ferroptosis in LSCC via activation of the mTOR pathway, underscoring the interplay between nutrient sensing, redox balance, and iron-dependent cell death [48]. Moreover, investigations by Zhao et al. have described a ferroptosis-related gene signature that stratifies LSCC patients by risk, further highlighting the potential for integrating ferroptosis profiling into clinical decision making [49]. Insights from broader head and neck oncology research support these findings. In other subtypes of head and neck squamous cell carcinoma, altered iron metabolism and sensitivity to ferroptosis have been linked to tumor growth and resistance to therapy, suggesting shared mechanisms that likely extend to LSCC. For instance, Lee and Roh emphasize that targeting iron uptake and storage pathways can enhance ferroptosis susceptibility [50], while Elhami et al. review ferroptosis induction as a promising strategy across oral, oropharyngeal, hypopharyngeal, and laryngeal cancers [51]. Collectively, these data suggest that iron metabolism in LSCC is rewired to support proliferation and suppress ferroptotic cell death, and that deliberate perturbation of this rewiring—for example, by increasing labile iron, inhibiting antioxidant defences, or targeting key iron transporters—may overcome resistance mechanisms and improve therapeutic outcomes. Given the overlapping roles of iron in oxidative stress, lipid peroxidation, and cellular metabolism, ferroptosis represents both a biological hallmark of metabolic reprogramming in LSCC and a novel therapeutic vulnerability with considerable translational potential (Table 4) (Figure 4).

## 7. Unique Metabolic Features of LSCC Compared with Other HNSCC Subtypes

Although HNSCC share several molecular and metabolic characteristics, emerging evidence suggests that LSCC exhibits distinct biological and metabolic features. Unlike oropharyngeal cancers, which are frequently associated with human papillomavirus infection and display specific immune and metabolic profiles, LSCC is predominantly linked to tobacco and alcohol exposure, resulting in different patterns of metabolic adaptation and microenvironmental interactions [52,53]. These differences are reflected in the metabolic landscape of LSCC, which appears to be more strongly associated with oxidative stress adaptation, lipid metabolism, and ferroptosis regulation. Chronic exposure to carcinogens and sustained inflammatory conditions may promote metabolic programs that enhance redox homeostasis and resistance to lipid peroxidation, thereby supporting tumor survival under stress conditions [5,11,45]. In addition, variations in TME composition and immune infiltration further contribute to distinct metabolic–immune interactions in LSCC. For instance, differences in lactate accumulation, glutamine utilization, and stromal engagement may influence immune suppression and therapeutic responsiveness in a manner that is not entirely shared with other HNSCC subtypes [9,13,17]. These observations suggest that LSCC should not be considered merely as part of a homogeneous HNSCC group, but rather as a biologically and metabolically distinct entity. Recognizing these differences is essential for the identification of subtype-specific biomarkers and for the development of tailored therapeutic strategies targeting metabolic vulnerabilities.

## 8. Discussion

Beyond individual metabolic alterations, LSCC metabolic reprogramming emerges as a highly interconnected network embedded within the TME, rather than a series of isolated pathways. The TME represents a dynamic ecosystem composed of tumor cells, immune populations—including cytotoxic T lymphocytes, regulatory T cells, tumor-associated macrophages, and myeloid-derived suppressor cells—and stromal components such as CAFs, endothelial cells, and extracellular matrix elements. These components engage in continuous bidirectional crosstalk, collectively shaping tumor progression, immune evasion, and therapeutic resistance [54]. Importantly, metabolic reprogramming acts as a key interface through which tumor cells modulate both immune and stromal compartments, linking intracellular metabolic adaptations to the functional organization of the TME.

Glycolysis, amino acid metabolism, lipid biosynthesis, and iron handling converge on shared functional outputs, including redox homeostasis, biosynthetic processes, and modulation of the TME. In this context, specific metabolites act as functional mediators linking tumor-intrinsic metabolic adaptations to stromal and immune responses. For instance, glycolysis-derived lactate not only reflects increased metabolic flux but also actively shapes the TME by promoting immunosuppression [13]. In LSCC, elevated lactate levels impair the cytotoxic activity of CD8^+^ T cells and NK cells while promoting the expansion of regulatory T cells, thereby linking tumor-intrinsic metabolic adaptation to immune evasion [9,17,18]. This highlights lactate as a functional mediator of tumor–immune crosstalk rather than a mere metabolic byproduct, with direct translational implications, as targeting lactate production or signaling may enhance the efficacy of immunotherapeutic strategies, including immune checkpoint blockade [9]. A similar level of integration is observed in amino acid metabolism, particularly in the context of glutamine utilization. In LSCC, glutamine metabolism is closely linked to mitochondrial function, where it provides carbon for the TCA cycle and supports nitrogen-dependent biosynthesis, such as nucleotide biosynthesis, and contributes to redox balance through NADPH production, especially under hypoxic and nutrient-limited conditions, as highlighted by Hou et al. [11]. These adaptations not only support tumor cell proliferation but also influence the TME by modulating oxidative stress and metabolic competition with immune cells. This metabolic dependency is further reinforced by molecular alterations such as the loss of CECR2, which enhances glutaminolysis and increases glutamine flux into mitochondrial and biosynthetic pathways. This shift not only supports tumor growth but also strengthens antioxidant defenses, thereby promoting adaptation to metabolic stress and contributing to therapeutic resistance. Importantly, restoration of CECR2 expression or pharmacological targeting of glutamine metabolism has been shown to impair tumor growth in preclinical models, underscoring the translational relevance of this metabolic axis [31]. Lipid metabolism further exemplifies the integration between metabolic rewiring and TME regulation, linking bioenergetics, inflammation, and therapeutic response. Early studies by Louw and Claassen [40] proposed that aberrant lipid utilization may contribute to radiotherapy failure and tumor recurrence by enhancing metabolic flexibility and resistance to oxidative stress, suggesting a potential role for lipid modulation as an adjuvant strategy. Beyond energy storage and membrane synthesis, lipid metabolites actively regulate inflammatory signaling within the TME, influencing the balance between pro- and anti-inflammatory mediators and contributing to an immunosuppressive milieu that supports tumor progression. These lipid-driven effects highlight how metabolic reprogramming extends to the regulation of tumor–stroma and tumor–immune interactions. Accordingly, the broad dysregulation of lipid biosynthetic and catabolic pathways in LSCC reinforces the therapeutic potential of targeting lipid metabolic nodes, such as fatty acid synthesis or uptake, to weaken tumor growth and enhance sensitivity to conventional treatments [39,41]. These metabolic adaptations converge with iron handling at the level of ferroptosis regulation, representing a critical intersection between lipid metabolism, redox balance, and cell death within the TME. LSCC cells frequently upregulate antioxidant systems, including GPX4 and components of the cystine/glutamate antiporter, to counteract iron-dependent lipid peroxidation and prevent ferroptotic cell death, thereby promoting survival under metabolic stress and resistance to conventional therapies. Conversely, pharmacological induction of ferroptosis through disruption of iron metabolism or lipid peroxidation defenses has emerged as a promising therapeutic strategy. In parallel, growing evidence supports the clinical relevance of ferroptosis in LSCC, as ferroptosis-related gene signatures have been shown to distinguish tumor from normal tissues, correlate with patient prognosis, and stratify patients according to risk and survival outcomes [45,46,47,48,49]. These findings further highlight ferroptosis not only as a metabolic vulnerability but also as a potential source of clinically actionable biomarkers.

Despite the growing body of evidence supporting the role of metabolic reprogramming in LSCC, several limitations and inconsistencies across studies should be acknowledged. Metabolic phenotypes may vary significantly depending on tumor stage, anatomical subsite, and microenvironmental conditions, leading to heterogeneous and sometimes conflicting findings across experimental models and patient cohorts. In particular, the relative contribution of glycolysis versus mitochondrial metabolism remains context-dependent, reflecting the high metabolic plasticity of tumor cells. Moreover, much of the current knowledge derives from in vitro systems and preclinical models, which may not fully recapitulate the complexity of the TME in vivo, thereby limiting the direct translatability of many proposed metabolic targets. While multiple metabolic pathways have been associated with immune modulation and therapeutic resistance, causal relationships are often inferred rather than validated in clinical settings. Significant gaps also remain in the identification of robust and reproducible metabolic biomarkers, as well as in understanding how intratumoral heterogeneity and dynamic metabolic adaptation influence treatment response.

Collectively, these findings highlight that metabolic and cellular components of the TME are tightly interconnected and mutually reinforcing, with central regulatory hubs—such as HIF-1α signaling, mitochondrial function, and redox balance—that integrate environmental constraints with intracellular metabolic adaptations. From a clinical perspective, these mechanisms may have important implications for treatment stratification and therapeutic decision-making. The identification of distinct metabolic phenotypes, including variations in glycolytic activity, glutamine dependency, and ferroptosis susceptibility, suggests that metabolic profiling could support patient selection and guide personalized therapeutic approaches.

In addition, several metabolic alterations discussed in this review hold promise as potential biomarkers for prognosis and treatment response, including ferroptosis-related gene signatures, metabolic subtypes, and regulators such as CECR2. Similarly, metabolite levels within the TME, such as lactate and lipid-derived mediators, may provide insights into tumor aggressiveness and immune status. However, the translation of these findings into clinical practice remains challenging due to tumor metabolic plasticity, intratumoral heterogeneity, and the dynamic nature of the TME, as well as the current lack of robust clinical validation. Addressing these limitations will require integrated approaches combining metabolic profiling, molecular characterization, and clinical data to identify reliable biomarkers and optimize therapeutic strategies.

## 9. Therapeutic Implications of Metabolic Reprogramming in LSCC

The growing understanding of metabolic reprogramming in LSCC provides a strong rationale for drug discovery and therapeutic innovation. Several metabolic pathways discussed in this review are potentially druggable targets, including key regulators of glycolysis, glutamine metabolism, lipid biosynthesis, and ferroptosis [5,11,45]. Targeting these pathways may impair tumor cell survival while simultaneously modulating the TME, and enhancing therapeutic efficacy [6,9]. In this context, drug development is likely to benefit from a biomarker-driven approach, in which metabolic phenotypes and molecular signatures are used to identify patient subgroups most likely to respond to specific interventions. For example, tumors characterized by high glycolytic activity and lactate production may be more sensitive to inhibitors of lactate transport or metabolism, particularly when combined with immune checkpoint blockade [9,13]. Similarly, glutamine-dependent tumors or those with altered ferroptosis regulation may be targeted through mitochondrial inhibitors or ferroptosis-inducing agents [11,45]. Importantly, the complexity and adaptability of tumor metabolism suggest that single-agent therapies may be insufficient, and that rationally designed combination strategies will be required [5]. These may include the integration of metabolic inhibitors with immunotherapy, radiotherapy, or targeted therapies, with the aim of simultaneously disrupting tumor metabolism and overcoming microenvironment-mediated resistance [6,7]. From a translational standpoint, early-phase clinical studies and broader HNSCC-oriented trials have begun to explore metabolism-targeting strategies, although LSCC-specific evidence remains limited. In parallel, metabolic imaging approaches such as 18F-FDG PET/CT are already clinically established in head and neck cancer, while emerging techniques may further refine metabolic characterization [55]. In addition, circulating biomarkers derived from biofluids represent a promising but still investigational tool for monitoring tumor metabolism and treatment response [56].

## 10. Challenges and Future Directions

Despite significant advances in understanding metabolic reprogramming in LSCC, several challenges limit the translation of these findings into clinical practice.

The high degree of metabolic plasticity, together with intratumoral heterogeneity and the dynamic nature of the TME, complicates the identification of stable and targetable metabolic vulnerabilities [5,9,11]. Moreover, the intricate crosstalk between tumor cells, immune populations, and stromal components makes it difficult to predict the effects of targeting single pathways, as compensatory mechanisms may rapidly emerge [6,7].

Another major challenge lies in the identification and validation of reliable metabolic biomarkers that can be applied in clinical settings. While emerging signatures, including ferroptosis-related genes and metabolic subtypes, show promise, their reproducibility and clinical utility remain to be confirmed in large, prospective cohorts [46,47,48,49,57,58]. In addition, most current evidence is derived from preclinical models, highlighting the need for studies that better recapitulate the complexity of human LSCC and its microenvironment [5].

In this context, the present review contributes to addressing these challenges by providing an integrated framework that connects metabolic pathways with TME interactions and immune modulation, moving beyond a pathway-specific perspective. By highlighting key metabolic hubs and their interplay with stromal and immune components, this work aims to facilitate the identification of clinically relevant biomarkers and to support the development of combinatorial therapeutic strategies. Such an approach may help overcome current limitations and guide future research toward more effective and personalized treatments in LSCC.

## 11. Conclusions

**Figure 5:**
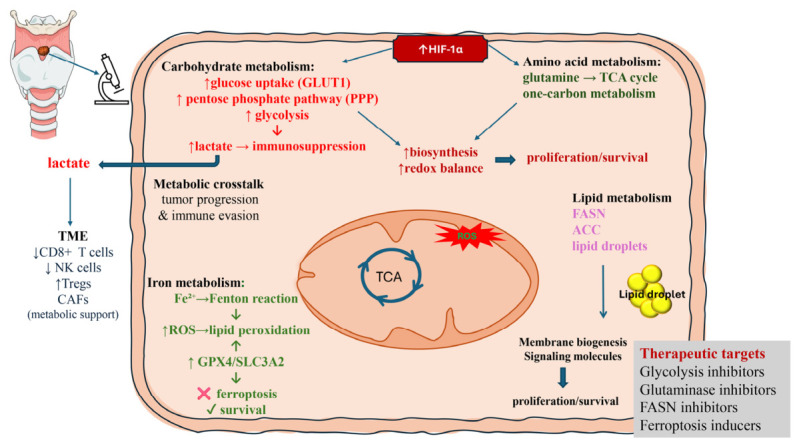
Integrated overview of metabolic reprogramming and tumor microenvironment interactions in laryngeal squamous cell carcinoma (LSCC). LSCC is characterized by extensive metabolic reprogramming involving glycolysis, amino acid metabolism, lipid metabolism, and iron handling, which are functionally integrated with the tumor microenvironment (TME). Under hypoxic conditions, HIF-1α acts as a central regulator, promoting glycolysis, pentose phosphate pathway (PPP), glutamine utilization, one-carbon metabolism, and lipid biosynthesis to sustain cellular proliferation, redox balance, and survival. Enhanced glycolysis leads to increased lactate production, which is exported into the TME and contributes to immunosuppression by impairing the function of CD8^+^ T cells and NK cells. Alterations in iron metabolism increase intracellular Fe^2+^ levels, driving reactive oxygen species (ROS) generation via the Fenton reaction and promoting lipid peroxidation; however, LSCC cells counteract ferroptosis through antioxidant and nutrient-sensing pathways, including GPX4, SLC3A2, thereby favoring tumor survival. Lipid metabolism supports membrane biogenesis and signaling, further contributing to tumor growth and microenvironmental interactions. Collectively, these interconnected pathways drive tumor progression, immune evasion, and metabolic crosstalk within the TME, while also representing potential therapeutic targets, including inhibitors of glycolysis, glutaminolysis, lipid synthesis, and ferroptosis-inducing strategies.

LSCC represents a paradigmatic example of how metabolic plasticity supports tumor progression, therapeutic resistance, and immune evasion. Rather than reflecting isolated alterations, metabolic changes in LSCC form an integrated and dynamic network that links tumor-intrinsic adaptations with microenvironmental and immune interactions (Figure 5). Increasing evidence indicates that these metabolic dependencies are not only central to tumor biology but also represent a source of clinically relevant vulnerabilities. In particular, the integration of metabolic profiling with molecular and microenvironmental characterization may improve patient stratification and support the development of more personalized therapeutic strategies.

From a translational perspective, targeting tumor metabolism is unlikely to be effective as a standalone approach, but may significantly enhance current treatment modalities when incorporated into rational combination strategies. However, the successful clinical implementation of these approaches will require robust validation in well-designed clinical studies, as well as the identification of reliable biomarkers capable of capturing tumor metabolic heterogeneity.

A more detailed characterization of these pathways, together with their interaction with immune and stromal components, may facilitate the identification of clinically relevant biomarkers and therapeutic targets. Future studies integrating metabolic profiling with molecular and clinical data will be essential to determine whether targeting metabolic dependencies can meaningfully complement existing treatment strategies in LSCC.

## Figures and Tables

**Table 1:** Key alterations of carbohydrate metabolism in cancer and LSCC.

Metabolic Pathway	Key Regulators	Physiological Role	Alterations in Cancer	Evidence in LSCC	Biological Implications
Glycolysis [12]	HIF-1α	ATP production from glucose	Increased aerobic glycolysis (Warburg effect)	Metabolomic evidence of enhanced glycolysis and lactate accumulation	Survival in hypoxic conditions, rapid energy supply
Mitochondrial OXPHOS [8]	Pyruvate flux regulation, mitochondrial activity	Efficient ATP generation	Preserved but functionally modulated	Mitochondrial function maintained	Metabolic plasticity and adaptability
PPP [5,14,15,16]	G6PD	NADPH production, nucleotide biosynthesis	Increased flux to support redox balance and biosynthesis	Increased G6PD activity in tumor tissue	Protection from oxidative stress, anabolic support
Lactate metabolism [17,18]	HIF-1α	Adaptation to low oxygen availability	Induction of glycolysis and suppression of mitochondrial oxidation	Indirect evidence from hypoxia-associated metabolic patterns	Oxygen-independent ATP production, reduced ROS
Hypoxia-driven metabolic regulation [9]	Lactate accumulation	End-product of glycolysis	Lactate acts as a signaling metabolite	Elevated lactate levels in the TME	Acidification, immune suppression, tumor progression

LSCC: laryngeal squamous cell carcinoma; OXPHOS: Oxidative Phosphorylation; PPP: Pentose phosphate pathway; HIF-1α: Hypoxia-inducible factors; G6PD: glucose-6-phosphate dehydrogenase; ATP: adenosine triphosphate; NADPH: Nicotinamide Adenine Dinucleotide Phosphate; ROS: Reactive Oxygen Species; TME: tumor microenvironment.

**Table 2:** Key alterations of amino acid metabolism in cancer and LSCC.

Metabolic Pathway	Key Regulators	Physiological Role	Alterations in Cancer	Evidence in LSCC	Biological Implications
Glutamine [11,23,24,31]	GLS, αKGDH, HIF-1α, SIAH2	Carbon and nitrogen source for TCA cycle, nucleotide synthesis, redox balance	Increased glutamine utilization; shift toward reductive carboxylation under hypoxia	Increased reliance on glutamine metabolism; regulation by CECR2	Supports proliferation, lipid synthesis, redox homeostasis, survival in hypoxia
Asparagine [25,26,27]	ASNS, LCK, NRF2	Amino acid homeostasis, signaling	Supports tumor growth; context-dependent immune modulation		Dual role in tumor survival and T cell-mediated immunity
Serine [28,29]	PHGDH, SHMT2, HIF-1α	One-carbon metabolism, NADPH production, nucleotide synthesis	Upregulation of serine synthesis and mitochondrial one-carbon pathway	Indirect evidence from metabolic rewiring	Antioxidant defence, epigenetic regulation, survival in hypoxia
Amino acid-TME interactions [22]	HIF-1α, immune signaling pathways	Metabolic-immune crosstalk	Amino acids act as signaling metabolites	Reviews report altered amino acid utilization in LSCC	Immune suppression, stromal remodeling, therapeutic resistance

LSCC: laryngeal squamous cell carcinoma; TME: tumor microenvironment; GLS: glutaminase; αKGDH: α-ketoglutarate dehydrogenase; HIF-1α: Hypoxia-inducible factors; SIAH2: Seven In Absentia Homolog 2; ASNS: asparagine synthetase; LCK: Lymphocyte-specific protein tyrosine kinase; NRF2: Nuclear Factor Erythroid 2–Related Factor 2; PHGDH: phosphoglycerate dehydrogenase; SHMT2: serine hydroxymethyltransferase 2; TCA: tricarboxylic acid; NADPH: Nicotinamide Adenine Dinucleotide Phosphate; CECR2: Cat Eye Syndrome Chromosome Region, Candidate 2.

**Table 3:** Key alterations of lipid metabolism in cancer and LSCC.

Lipid Pathway	Key Regulators	Physiological Role	Alterations in Cancer	Evidence in LSCC	Biological Implications
De novo fatty acid synthesis [32,33,39]	ACC, FASN, SREBP	Membrane biogenesis, energy storage	Upregulation of lipogenic enzymes	Overexpression of ACC and FASN	Enhanced proliferation, membrane remodeling
Hypoxia-driven lipid reprogramming [34,35]	HIF-1α	Metabolic adaptation to low oxygen	Increased lipid synthesis and lipid droplet accumulation	Hypoxia-associated lipid rewiring	Survival under hypoxic stress, protection from lipotoxicity
Lipid uptake and transport [39,40]	FABPs, CD36	Fatty acid uptake and trafficking	Upregulated lipid import	Increased expression in LSCC	Metabolic flexibility, energy supply
Lipid-mediated immune and inflammatory signaling [41]	Eicosanoids, lipid mediators	Immune modulation	Dysregulated lipid-derived signaling	Altered inflammatory milieu	Immune suppression, tumor–stroma crosstalk

LSCC: laryngeal squamous cell carcinoma; ACC: acetyl-CoA carboxylase; FASN: fatty acid synthase; SREBP: sterol regulatory element-binding protein; HIF-1α: Hypoxia-inducible factors; FABPs: fatty acid binding proteins.

**Table 4:** Key alterations of carbohydrate metabolism.

Pathway	Key Regulators	Physiological Role	Alterations in Cancer	Evidence in LSCC	Biological Implications
Iron uptake and storage [44,50]	Transferrin, ferritin, iron transporters	DNA synthesis, oxygen transport, mitochondrial function	Increased iron uptake; altered storage; labile iron pool expansion	LSCC cells exhibit dysregulated iron homeostasis	Supports proliferation; creates potential for ROS-mediated damage
Ferroptosis [45,46,47,48,49]	Lipid peroxidation, GPX4, cystine/glutamate antiporter (SLC3A2)	Iron-dependent regulated cell death	Tumor cells upregulate antioxidant systems to prevent ferroptosis	Upregulation of GPX4, SLC3A2; ferroptosis-related gene signatures correlate with prognosis	Dual role: vulnerability vs. tumor survival; therapeutic target
Lipid peroxidation [45]	Polyunsaturated fatty acids, ROS	Mediates ferroptotic death	Increased susceptibility under high iron and oxidative stress	Modulated by antioxidant defences	Modulates sensitivity to therapy and cell death
Iron-targeted therapy [50,51]	Pharmacological inducers of ferroptosis	Exploit iron-dependent cell death	Preclinical studies show enhanced tumor cell death	Proposed as non-surgical strategy in LSCC	Potential therapeutic avenue; may overcome resistance

LSCC: laryngeal squamous cell carcinoma; GPX4: glutathione peroxidase 4; SLC3A2: Solute Carrier Family 3 Member 2; ROS: Reactive Oxygen Species.

## Data Availability

No new data were created or analyzed in this study. Data sharing is not applicable to this article.

## Abbreviations

The following abbreviations are used in this manuscript:

LSCC	Laryngeal Squamous Cell Carcinoma
TME	Tumor Microenvironment
HNSCC	Head and Neck Squamous Cell Carcinoma
CAFs	Cancer-Associated Fibroblasts
NK	Natural Killer
HIF-1α	hypoxia-inducible factor 1
ATP	adenosine triphosphate
HIFs	Hypoxia-inducible factors
NADPH	Nicotinamide Adenine Dinucleotide Phosphate
FAO	fatty acid oxidation
TCA	tricarboxylic acid
PPP	pentose phosphate pathway
NADH	Nicotinamide Adenine Dinucleotide
G6PD	glucose-6-phosphate dehydrogenase
ACLY	ATP-citrate lyase
H3K18la	histone H3K18 lactylation
RUNX2	Runt-related transcription factor 2
GLS	glutaminase
α-KG	α-ketoglutarate
αKGDH	α-ketoglutarate dehydrogenase
SIAH2	Seven In Absentia Homolog 2
UDP-GlcNAc	uridine diphosphate N-uridine diphosphate N-acetylglucosamine
Asn	Asparagine
Asp	aspartate
ASNS	asparagine synthetase
LCK	lymphocyte-specific protein tyrosine kinase
NRF2	Nuclear Factor Erythroid 2–Related Factor 2
SHMT2	serine hydroxymethyltransferase 2
CECR2	Cat Eye Syndrome Chromosome Region, Candidate 2
ACC	acetyl-CoA carboxylase
FASN	fatty acid synthase
SREBPs	sterol regulatory element-binding proteins
LDs	lipid droplet
SCD1	stearoyl-CoA desaturase-1
GPX4	glutathione peroxidase 4
SLC3A2	Solute Carrier Family 3 Member 2
